# Advances in Engineered Metal Oxide Thin Films by Low-Cost, Solution-Based Techniques for Green Hydrogen Production

**DOI:** 10.3390/nano12121957

**Published:** 2022-06-07

**Authors:** Ingrid Rodríguez-Gutiérrez, Karen Cristina Bedin, Beatriz Mouriño, João Batista Souza Junior, Flavio Leandro Souza

**Affiliations:** 1 Centro de Ciências Naturais e Humanas (CCNH), Federal University of ABC (UFABC), Santo André 09210-580, SP, Brazil; 2 Brazilian Nanotechnology National Laboratory (LNNANO), Brazilian Center for Research in Energy and Materials (CNPEM), Campinas 13083-970, SP, Brazil; karen.bedin@lnnano.cnpem.br (K.C.B.); beatrizmourinoap@gmail.com (B.M.); joao.junior@lnnano.cnpem.br (J.B.S.J.); 3 Institute of Chemistry, University of Campinas (UNICAMP), P.O. Box 6154, Campinas 13083-970, SP, Brazil

**Keywords:** iron oxide, solution chemistry, photoanodes, hydrothermal synthesis, sol-gel method

## Abstract

Functional oxide materials have become crucial in the continuous development of various fields, including those for energy applications. In this aspect, the synthesis of nanomaterials for low-cost green hydrogen production represents a huge challenge that needs to be overcome to move toward the next generation of efficient systems and devices. This perspective presents a critical assessment of hydrothermal and polymeric precursor methods as potential approaches to designing photoelectrodes for future industrial implementation. The main conditions that can affect the photoanode’s physical and chemical characteristics, such as morphology, particle size, defects chemistry, dimensionality, and crystal orientation, and how they influence the photoelectrochemical performance are highlighted in this report. Strategies to tune and engineer photoelectrode and an outlook for developing efficient solar-to-hydrogen conversion using an inexpensive and stable material will also be addressed.

## 1. Introduction

Global warming and non-natural environmental disasters are obvious evidence of the harmful effects of climate change that is driven by anthropogenic activities, which mainly originate from the uncontrolled use of fossil fuels to meet the world’s energy demands. Realizing this, nearly 200 countries signed legally binding international treaties in the Paris Agreement to reduce greenhouse gas emissions and to control the rise in global temperature [1]. In 2021, this commitment was renewed with even more ambitious climate goals during the 26th Conference of the Parties (COP 26). The agreement established a gradual reduction in subsidies for fossil fuels and coal use, and established rules for the carbon credits market among countries [2,3]. In this context, carbon-neutral or emissions-free energy sources are imperative for achieving these goals and an environmentally sustainable society.

The key to developing or establishing new technologies and benchmarks in energy research has been based on nanomaterials [4,5]. Hence, nanotechnology will play an essential role in transitioning from fossil fuels to renewable, sustainable, and clean energy. Among the promising technologies for energy production, hydrogen (H_2_) obtained from solar water splitting has gained considerable attention [6,7,8,9]. According to the International Energy Agency (IEA), a significant barrier that limits the development of clean hydrogen industry is related to the existing regulations for H_2_ production, storage, and transport [10]. Several countries, including Australia, China, Brazil, Chile, Finland, Germany, Norway, Portugal, Spain, the United States, and the European Union, adopted different strategies for reaching the net-zero emissions goal by 2050. The joint strategies between developing and developed countries have been prominently featured in the agenda of green hydrogen and economic transition. In this context, several consortiums and multi-million investments were also created for green hydrogen research and technologies [11,12,13,14]. Unfortunately, ongoing efforts have not successfully produced materials with a solar-to-hydrogen conversion (STH) efficiency suitable for application in photoelectrochemical (PEC) devices. Therefore, only a few companies still believe in the technology’s viability for supplying the energy demand [13,15]. Hence, producing hydrogen from this low-carbon approach has become a great challenge that requires international cooperation to be extended to scientific, industrial, and political areas.

**Figure 1:**
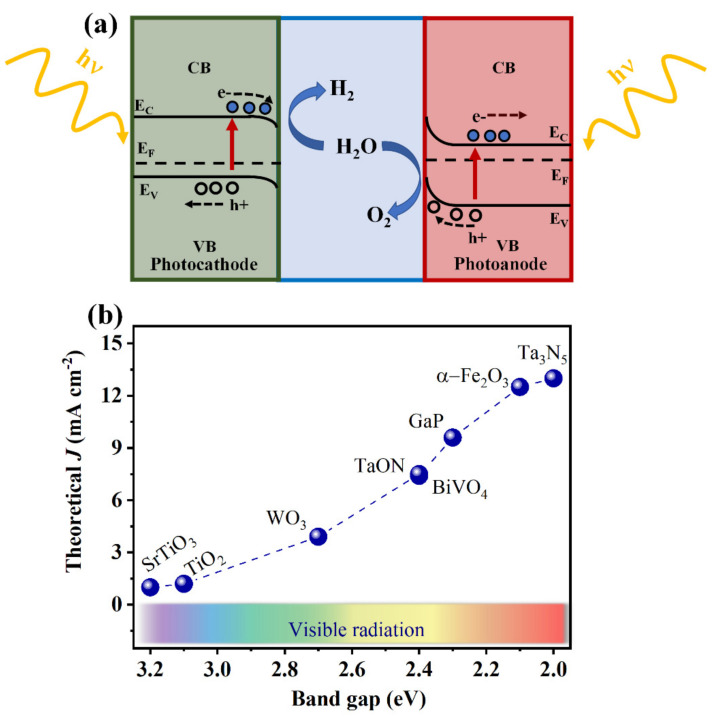
(**a**) Schematic of the main design aspects for a PEC design. Ec and Ev represent the conduction band (CB), valence band (VB) edges, and E_F_ represents the Fermi level. (**b**) Theoretical photocurrent density (J) values towards sunlight-driven water splitting for n-type semiconductors, materials, and their bandgap energy.

Over the years, several groups have reported the main principles of PEC devices, their possible configurations, and their functionality [16,17,18,19]. In general, a PEC device is composed of two electrodes, of which at least one is fabricated with a photoactive functional material (photoelectrode). The goal of photoelectrocatalytic water splitting is to create a PEC device (Figure 1a) composed of two photoactive materials, a photoanode and a photocathode, which is capable of generating electron-hole pairs from direct sunlight absorption to split the water molecule in the most cost-efficient way. These photoelectrodes can capture the sunlight energy and produce gaseous O_2_ at the photoanode and H_2_ at the photocathode, as observed in Figure 1a. The efficiency of a PEC device is mainly associated with the photoanode’s photocurrent, since the oxygen evolution reaction is the limiting step due to the necessity of transferring four holes to produce O_2_ [16,17]. Compared with a typical electrolyzer, a low overpotential (η) over the thermodynamic potential of water splitting (1.23 V vs. NHE) is needed to drive the PEC water splitting. This overpotential in photoanodes is due to the energy losses related to the photoholes passing through the space charge region and to the electron flow through the external circuit to the counter electrode [20,21]. Taking into account the thermodynamic and kinetic energy losses (~0.7–1.0 eV), both can be compensated in the minimum required bandgap. Developing functional and efficient photoanodes using abundant elements with long-term stability, reproducibility, and that can be prepared by scalable methods, which can be easily integrated by industries is crucial to make PEC technology the main alternative for green hydrogen production. 

An ideal photoanode must possess a suitable bandgap (~1.8 eV due to the η) to efficiently absorb the natural sunlight and the band positions must be aligned with the thermodynamic potential of water oxidation (1.23 V vs. NHE). It is expected that in an ideal photoanode candidate, after light absorption and the subsequent generation of photocarriers, the electron-hole pairs will separate efficiently and migrate to the back contact or to the surface, respectively, without recombining. Finally, the photoanode must have a strong catalytic efficiency and great stability [21]. Figure 1b shows the main candidates as photoanodes that meet these requirements by comparing their correspondent theoretical photocurrent (J) with their bandgap (E_g_) function. It can be noted that most materials are metal oxides with different E_g_ in the visible light spectrum—from violet at ~400 nm, to red light at ~700 nm. In particular, those a with wide E_g_ (>2.6 eV) have a considerable limitation on their maximum photocurrent density, demanding modifications or combinations with other materials to increase their PEC performances. Furthermore, it is essential to consider a minimum STH of 10% for commercial purposes that correspond to ~8.13 mA cm^−1^, according to equation 1, where η_F_ represents the faradaic efficiency and P the incident light power. It is worth mentioning that this photocurrent density can be generated only from a semiconductor with an E_g_ smaller than ~2.3 eV [22].

(1) $$\mathrm{STH}={\left[\frac{\left|J\left(\frac{\mathrm{mA}}{{\mathrm{cm}}^{2}}\right)\right|\times 1.23\;\left(V\right)\times \eta_{F}}{P\left(\frac{\mathrm{mW}}{{\mathrm{cm}}^{2}}\right)}\right]}_{\mathrm{AM}\;1.5G}$$

The choice of photoactive materials to compose the PEC system is limited. Considering the materials displayed in Figure 1b, BiVO_4_ and TaON have a bandgap of 2.4 eV, which hampers their maximum performance. TaON can be easily oxidized in aqueous media containing oxygen [23]. In addition, BiVO_4_ is susceptible to corrosion and photocorrosion in neutral and alkaline solutions, and toxic intermediates might be generated from these processes [24,25]. Similarly, GaP and Ta_3_N_5_ suffer photocorrosion and surface oxidation in alkaline media, resulting in poor stability toward the water splitting reaction, which limits their application to only a few minutes without any protective surface layer [26,27,28,29]. Other semiconductors, such as III-V photoanodes [30,31,32,33,34] or a doped BiVO_4_ scheelite structure [35,36,37], have shown interesting results. Still, their fabrication costs, photocorrosion, and instability issues in aqueous solution make them unsuitable for water splitting. On the other hand, hematite (α-Fe_2_O_3_) is non-toxic, exhibits greater stability, and is abundant on Earth (and Mars). For these reasons, it has been widely studied as a photoanode towards water splitting induced by sunlight. Nevertheless, hematite, by itself, suffers several shortcomings that limit its photoelectrochemical performance; its relatively low absorption coefficient and indirect-semiconductor nature typically requires a film thickness of ~400–500 nm for full visible-light absorption [38]. Moreover, its inherent short hole diffusion length (2–4 nm) and its minority carrier lifetime restricts the efficient charge collection of them via the interfacial charge-transfer reactions [39,40]. The presence of surface states that act as recombination centers also hinders the hematite’s PEC response [40,41,42,43]. In addition, hematite also possesses a rather poor electron conductivity (~10^−2^ cm^2^ V^−1^ s^−1^), which can only be improved by doping to increase its electron density and conductivity [44].

**Figure 2:**
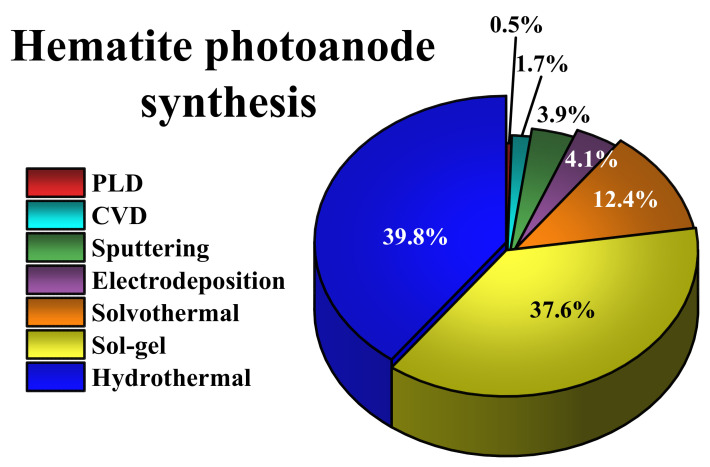
Publications found in the Scopus database, using the search term “hematite photoanode synthesis” and the topics enlisted in the legend from 2000 to 2021 (as of January 2022).

In order to overcome the problems mentioned above, several strategies for improving hematite’s electronic transport, optical properties, and charge carrier dynamics have been widely explored. For instance, nanostructuring [45,46,47], surface modifiers [48,49,50], and dopant addition [51,52,53], among others, have been reported and reviewed in the literature to derive synergic approaches capable of increasing the photocurrent benchmarks closer to the theoretical values, and to accomplish the industry-required STH efficiency. Moreover, various fabrication methods have also been developed for increasing this benchmark, including pulsed laser deposition (PLD) [54,55], reactive sputtering [56,57,58], chemical vapor deposition (CVD) [59,60], electrodeposition [61,62,63,64], solvothermal [65,66,67], hydrothermal [68,69,70,71,72], and sol-gel-based approaches [73,74,75,76]. From a simple search in the Scopus (Elsevier) database, displayed in Figure 2, it can be seen that hydrothermal and sol-gel synthesis is involved in ~80% of the reports, whereas others only represent ~20%. Using techniques such as PLD or CVD is useful academically because they allow the controlled deposition of a thin oxide layer onto conductive substrates; however, their high costs prohibit the large-scale manufacturing of large samples.

On the other hand, solution-based fabrication techniques—such as electrodeposition, solvothermal, hydrothermal, and sol-gel—are indeed cost-effective and already implemented in various key industry sectors. They should be environmentally friendly to maintain the sustainability of the PEC hydrogen produced. Solvothermal methods that use non-aqueous precursor solutions and electrodepositions in organic solvents are no longer attractive for large-scale manufacturing. Therefore, hydrothermal and sol-gel-based approaches demonstrate more significant potential for industrial implementation to fabricate many nanostructured oxides onto various substrates with engineered morphologies, dimensionalities, and thicknesses. The sol-gel method encompasses the preparation of precursors in different chemical environments and has been used indiscriminately in the area. Excellent stoichiometric control, reproducibility, and versatility are particularly achieved by the polymeric precursor method, a strand of the sol-gel field. In this perspective, both fabrication methods will be addressed towards a benchmark design, using a hematite photoanode as a case study and a low-cost material; we will also highlight the recent progress and the challenging aspects that still limit its commercial application. Later in this report, we will discuss our latest group achievements and the results that might provide a favorable route to designing efficient nanomaterials for photoelectrochemical and optoelectronic applications, among others. We will also express our opinions on points that should be explored in the future in order to fabricate engineered materials by low-cost and straightforward methods, to make hydrogen production (i.e., green H_2_) from solar water splitting a sustainable process. 

## 2. Hydrothermal Synthesis

Hydrothermal synthesis of functional materials can be conventionally performed in a wide temperature range, from room temperature to very high temperatures [77]. Since the first application of the hydrothermal process for nanomaterial synthesis in the 1990s, significant advances in understanding the physical–chemical features of this synthesis have been made [78]. As will be discussed in this section, the main parameters of the hydrothermal synthesis (i.e., initial pH of the precursor solution, duration, and temperature) define the process kinetics and the properties of the products, which are directly related with pressure in the system [79]. The energy-conserving hydrothermal conditions favor the crystallization of high-purity powders, with few point defects and allow the recovery of the chemicals used in the process [80,81]. From a technological and commercial viewpoint, these aspects that are associated with low energy consumption superimpose over the similar solvothermal synthesis, due to the absence of a non-aqueous solvent or a surfactant to assist the chemical reactions. Thus, hydrothermal synthesis represents an environmentally friendly option for metal oxide synthesis, both in powder and film form [82,83,84].

In a typical metal oxide hydrothermal synthesis, the solution is composed of a metal cation precursor, an ionic strength controller, and of water as a solvent. A substrate is added to the system when desired to prepare nanostructured oxide films. The synthesis is carried out under subcritical water conditions in an autoclave reactor, generating an autogenous pressure and changing the physical–chemical water properties. Due to the water viscosity and the high ionic strength, hydrolysis reactions are favored without any catalyst. Traditionally, this effect is more pronounced closer to the critical point of water (374 °C and 22.1 MPa) [85,86,87]. 

Notwithstanding the complexity involved in such equilibria, many inorganic compounds have their solubility in aqueous media calculated by thermodynamic models, such as the Helgeson–Kirkham–Flowers (HKF) model, which other researchers have already reviewed to obtain more accurate calculations [86]. Considering that the oxide surface in aqueous media follows the Brønsted–Lowry theory, a net charge density is always present when the solution pH is away from the point of zero charge (zero net charge density or PZC), and the interfacial tension is reduced [88]. It has been shown that the proton adsorption at the surface induces the lowering of the surface/interfacial tension (dγ < 0). This reduction also occurs by controlling the pH and increasing the ionic strength, kinetically favoring the precipitation of the less thermodynamically stable allotropic and high soluble phase [71]. As such, in some cases, sintering steps may not be necessary since an intermediate or the preferable metal oxide phase can be directly obtained from hydrothermal conditions. The crystallized product formation drives the nucleation rate, the particle growth, and the aging processes in the dissolution–recrystallization regime that governs the synthesis [80]. Therefore, it is evident that nanoparticles’ morphology, size distribution, and crystallographic phase/direction in powder and thin films can be delineated by controlling the experimental parameters. 

The main disadvantages of the hydrothermal method are the high cost of the equipment and the inability to monitor crystal growth during the process. To avoid the usage of complex hydrothermal reactors and to decrease energy consumption, an interesting modification from the typical hydrothermal synthesis, which involves low temperatures and low pressures, was developed [71]. This synthesis, called “purpose-built materials”, was based on the idea of monitoring the thermodynamics and kinetics of nucleation and growth by experimentally controlling the interfacial tension. Such control allows the ability to separate the nucleation and the growth stage, which generates monodisperse nanoparticles with narrow size distribution. As the precipitation occurs far from the typical PZC of the metal oxide, the presence of charged surface sites is major, as it contributes to the further lowering of the interfacial tension of the system. 

In essence, the method of the purpose-built material can be used to prepare any metal oxide with any morphology. The synthesis of nanostructured thin films by purpose-built materials involves an ideal simultaneous control of different aspects [89,90,91]. The first is associated with structural control, since the precipitation of nanoparticles at very low interfacial tension leads to the thermodynamic stabilization of metastable crystal structure. Another aspect is related to morphology control, which is also reached by the thermodynamic stabilization of the anisotropic morphology. The last is associated with the orientation control that is principally achieved by considering the difference of interfacial energy between the substrate and the nanoparticles, which preferentially allows nucleation and growth onto the substrate (heteronucleation) rather than in the solution (homogeneous nucleation). Chemically induced (i.e., pH, ionic strength), very slow kinetics of nucleation and growth allows the anisotropic nanoparticles to grow perpendicularly to the substrates to form highly oriented nanorod arrays. Faster kinetics enables the growth of nanorods with parallel orientation onto the substrates [92]. An extended explanation can be found in [90]. The perpendicularly oriented nanorod arrays enabled us to overcome the limited hole diffusion length by matching it with the diameter of the rods and by providing grain-boundary free pathways for the photogenerated electrons to reach the conducting substrate. This substantially reduced the electron-hole recombination, thus reaching an incident photon-to-electron conversion efficiency (IPCE) of 56% at 340 nm (i.e., more than half of the incident photons are collected as electrons) in a 2-electrode (sandwich) cell. Moreover, confinement effects in ultrafine hematite nanorods have been reached to shift the conduction band edge upward (0.3–0.6 eV) to obtain hydrogen generation capability (at the Pt cathode) without any applied bias [91]. 

**Figure 3:**
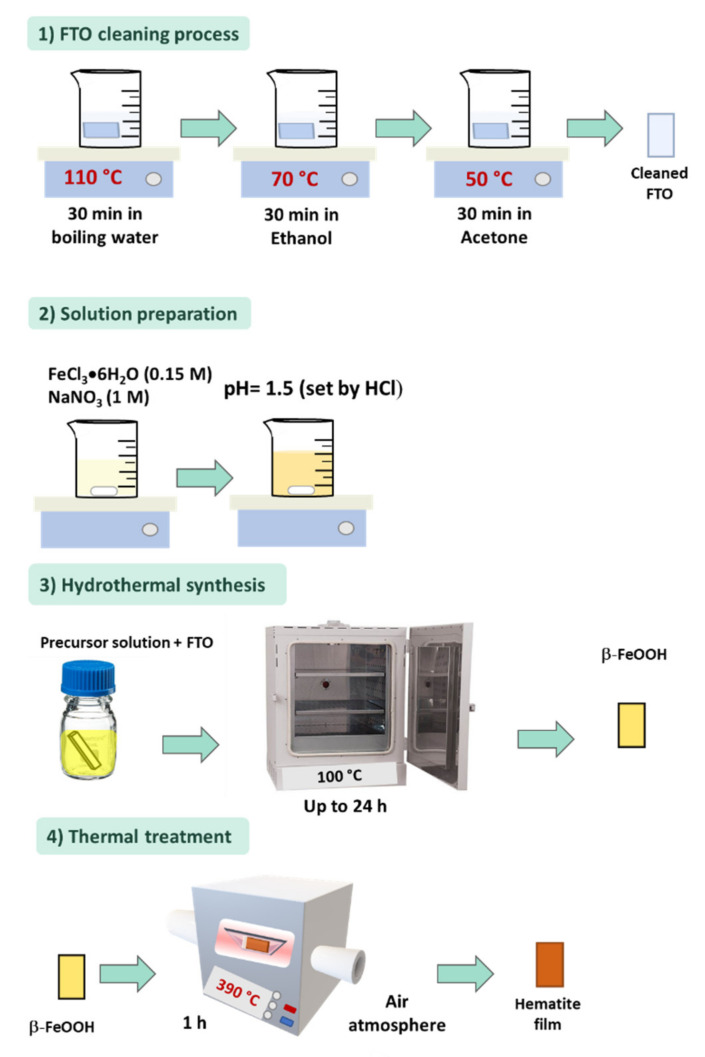
Experimental procedure for the synthesis of hematite nanorod arrays by the purpose-built materials route. Fluorine-doped tin oxide (FTO) glass substrate corresponds to the commercial substrate. Solution preparation represents the complete FeCl_3_ and NaNO_3_ dissolution and, subsequently, the pH adjustment, at 1.5. The pioneer report [51] performed the hydrothermal growth for up to 24 h. Lastly, the as-synthesized film was thermally treated at 390 °C for 1 h.

Considering that the nanorod morphology can help to overcome most of the electronic drawbacks of hematite as a photoanode, this section will discuss the synthesis of hematite nanorod arrays by purpose-built materials methodology. Figure 3 illustrates the experimental procedure employed to obtain iron-based nanostructured films from the hydrothermal approach. As illustrated, this simple procedure can be divided into four essential steps, which directly influence the quality of the film. The first one is related to substrate cleaning. Although this step is usually underrated, it must be carefully performed since the presence of any particle in the substrate could obstruct the uniform growth. The second step involves the solution preparation, principally composed of 0.15 M of FeCl_3_, 1 M of NaNO_3_, and pH adjustment at 1.5. Considering that this methodology contemplates the precipitation far from the PZC, a high concentration of precursors, a low pH, and a high ionic strength, combined with a low temperature constitutes a perfect formula for nanorod array formation [89,92,93]. Some modifications in this solution by substituting NaNO_3_ with other salts to control the ionic strength in the same hydrothermal conditions tend to form other nanostructures, such as nanowires or nanocubes [94]. 

During the hydrothermal procedure (Figure 3, third step), the thermodynamics and kinetics of nucleation and growth processes onto glass substrates play an essential role in nanoparticle design. Fortunately, since this growth occurs at a relatively low temperature (100 °C) and low pressure, a regular glass flask can be employed as a reactor to design nanostructures and thin films. Furthermore, other reports have shown that varying the reaction time does not affect the nanorod morphology but tunes the nanorod length and diameter. However, when hydrothermal synthesis is performed at higher temperatures, different morphologies can be seen, such as a spherical shape [85]. 

The last, but not least important step in designing hematite nanorod arrays corresponds to the thermal treatment. At the end of the hydrothermal synthesis, a β-FeOOH layer deposited onto a substrate (usually a transparent conductive oxide substrate, such as fluorine-doped tin oxide, FTO) is obtained. During the thermal treatment, a crystal phase transformation to hematite occurs [95,96]. Even though this step can be performed at 390 °C, similarly to the original route, several reports have demonstrated the need for higher temperatures to evolve the phase transformation and activate the hematite phase. The thermal treatment at different temperatures and atmospheres plays a vital role in the PEC performance, since it can modulate defect chemistry, induce Sn self-doping, and activate the material. However, the substrate can be damaged in this step, thus significantly impacting the PEC performance and reproducibility [97]. After the thermal treatment, some interface mismatches are also created [98]. Therefore, the optimization of this process is essential to enhance the hematite PEC performance and to minimize substrate damage [70,99,100,101,102]. Some studies have been devoted to understanding how the thermal treatment influences the hematite electronic structure, the chemistry of defects in hematite, FTO, and the hematite–FTO interface, as well as the substrate damage caused by the element diffusion from FTO to hematite. However, there are contrasting ideas on this matter [96,101,103,104,105].

**Figure 4:**
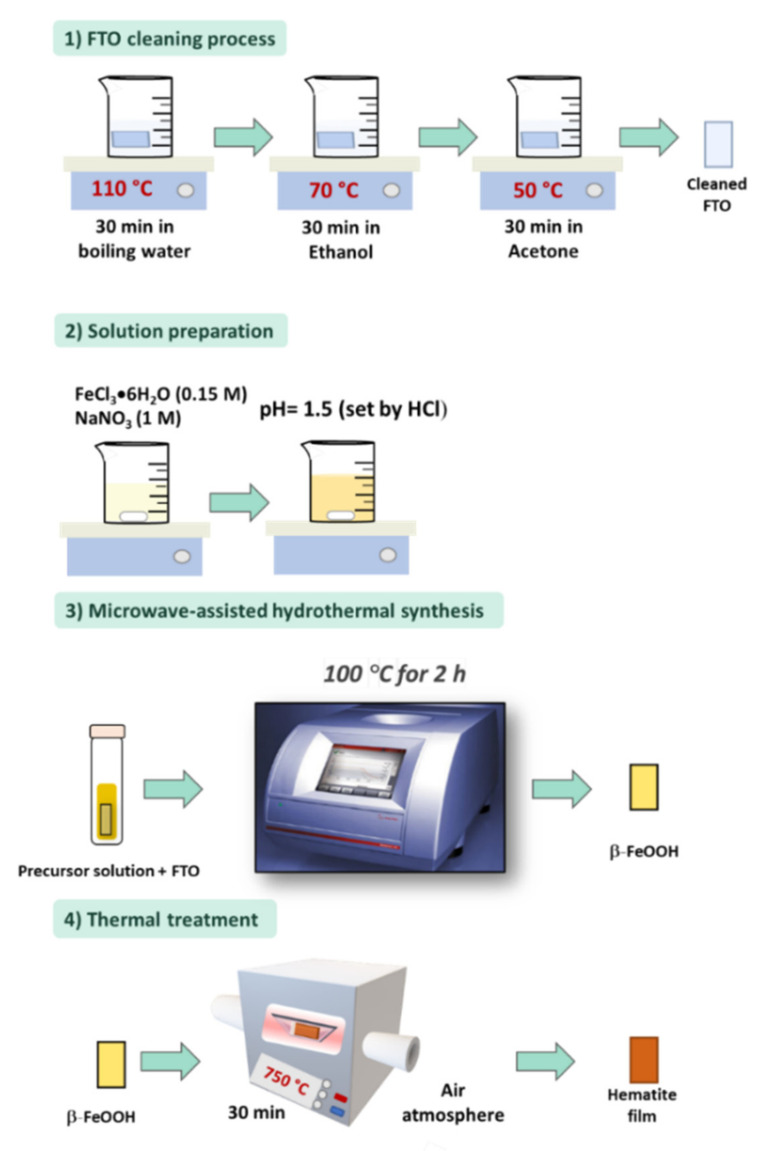
Experimental procedure for microwave (MW) hydrothermal synthesis of hematite nanorod arrays by purpose-built materials route. A precursor solution used for hydrothermal synthesis, consisting of FeCl_3_ and NaNO_3_ dissolution and pH adjusted at 1.5. The as-synthesized film is thermally treated at 750 °C for 30 min to obtain hematite films.

An attractive adapted methodology proposed in 2015 [106] was to induce film growth by employing the microwave-assisted (MW) hydrothermal route (Figure 4). The authors departed from the same precursor solution mentioned above and used a commercial microwave reactor to synthesize the films for 2 h. In microwave-assisted synthesis, the reaction is dependent on the interaction between the microwave and the precursor solution. The solvent is polarized with the electromagnetic field, promoting more uniform and faster heating than conventional hydrothermal synthesis [107]. Due to this change, a β-FeOOH nanorod array film with a thinner thickness than the original procedure was obtained. The authors also showed that the atmosphere during the thermal treatment plays a vital role in creating defects or oxygen vacancies that modify the hematite electronic properties and influence the PEC response. This approach has recently been used for synthesizing hematite films modified with Zr, with promising results [108].

The advantage of using these hematite nanostructured arrays on PEC water splitting lies in overcoming its main drawback as a photoanode, which is its very limited hole diffusion length. In addition, the high surface area provides a large region that is in contact with the electrolyte, which is favorable for the charge carrier kinetics [73,109]. Nanoparticles and mesoporous films suffer from high bulk recombination and poor charge transport, mainly related to the high number of grain boundaries found in these morphologies [110,111,112,113]. In contrast, morphologies oriented in the [110] direction have higher anisotropic conductivity, resulting in an improved charge carrier collection by minimizing the hopping transport and reducing the recombination losses at grain boundaries [70,114,115]. Morphologies—such as nanowires, nanotubes, and nanorods—can reduce the distance needed for hole transfer, reduce electron-hole recombination, and overcome transport limitations. 

Considering that surface states influence hematite PEC performance, Wang et al. studied the PEC performance of hematite nanostructured arrays in nanocubes, nanowires, and nanorods [94]. This study showed that the bandgap decreases in the sequence of nanowires, nanorods, and nanocubes. The authors ascribe the PEC of hematite nanocubes to its superior carrier density number and to the anodically shifted surface states, leading to a higher onset potential. However, nanocubes’ light absorption is limited due to the compact arrangement of nanocubes, which diminishes the number of photogenerated charges. In contrast, the lower surface recombination influences the nanowire array response, despite the unfavorable light absorption. Otherwise, nanorod arrays were the only morphology that showed improved light absorption, contributing to higher charge carrier photogeneration. However, the rod diameter can lead to a more significant overall recombination than the nanowires, affecting their PEC performance. 

The higher light absorption demonstrated by the hematite nanorod arrays with an optimized diameter could potentially address some of the fundamental PEC issues [116]. Although the nanorod arrays report improved photocurrent density, the photoresponse was much lower than the theoretical response. Improving the photoresponse by doping hematite structures with a preferred orientation has become a significant challenge in the field. Adding elements [53,96,113,117,118,119,120,121,122,123,124,125,126,127] such as Ti, Zr, Sn, Sb, Zn, Ge, Nb, and Ta into the hydrothermal precursor solution facilitates the formation of a hematite-doped structure, since the FeOOH precursor presents a higher donor density [128]. However, increasing the donor density number usually provokes a reduction in the width of the space charge region, which also affects the PEC performance.

On the other hand, it has been shown that the deposition of an overlayer onto a β-FeOOH nanorod array and subsequent thermal treatment can produce even better PEC results than the precursor solution modification [129,130,131]. Independent of the strategy of element addition, the literature has associated the hematite performance with the increase of the donor density number, owned by the doping of the hematite structure. In a recent contribution [53], we have shown experimental evidence that the overlayer strategy leads to the segregation of the elements at the grain boundaries, which lowers the energy barrier at the boundaries and facilitates electron collection. Overlayer deposition usually involves the creation of additional surface states that shift the photocurrent onset potential to more positive values. Consequently, the necessity of modifying the surface by adding a surface passivating agent, plasmons, or a cocatalyst has been highlighted [49,132,133,134,135,136]. Later studies showed that a combination of these strategies achieved the photocurrent record reported to date: the hydrothermal synthesis of a β-FeOOH nanorod array; a subsequent deposition of a TiO_2_ overlayer, followed by thermal treatment; and, subsequently, Co-Pi electrodeposition as a surface modifier, as shown by Jeon et al. [137], which reached an outstanding performance of 6.0 mA cm^−2^ at 1.23 V vs. RHE. Compared with the bare photoanode, this system also showed good stability, enhanced charge separation efficiency, and catalytic efficiency (η_Sep_ and η_Cat_, respectively). 

The responses accomplished by nanorod array films represent a step forward in designing highly efficient devices for solar water splitting. Their scale-up restrictions are the main complication for them being used on an industrial scale. Alternative methods that are more suitable for industrial application have commonly led to the formation of multilayer oxide films, with different morphologies (e.g.,: spheres, cubes, ellipsoidal nanoparticle shapes, etc.) instead of the promising, vertical-aligned columnar morphology. This multilayer film presents a huge number of interfaces or grain boundaries, which have hindered the high efficiency photoanodes due to the high recombination rate or electron trapping at those interfaces. For instance, sol-gel, an industry-friendly method, has been pursued to produce photoelectrodes, such as hematite. The scientific evolution of this method over the decades has partially mitigated those interfacial problems, achieving efficiency comparable to the one-dimensional morphology. In this context, our group has optimized and adapted the sol-gel methods to engineer a high-efficiency hematite photoanode, as described in the next section. 

## 3. Polymeric-Precursor Solution-Based Method

The sol-gel method is an alternative approach to synthesizing functional nanomaterials, such as powder, suspensions, and thin films. The term “sol-gel” generally consists of a gradual transition from liquid precursors to a colloidal suspension, sol, and then to a gel-like network to finally obtain inorganic polymers or ceramics [138,139]. These materials have a wide range of applications that cover daily use, such as in windows and porcelain for households, and advanced technologies, such as airplanes, rockets, smartphone batteries, and catalysts for alternative energies.

Kakihana [140] divided sol-gel technology into the following three categories, according to the precursor gel: (a) colloidal sol-gel; (b) inorganic polymeric gel, derived from organometallic compounds; and (c) gel routes involving the formation of organic polymeric glass. The last category that is also known as the polymeric precursor (PP) route, is based on the formation of very stable and water-soluble metal-chelate complexes that react with a polymerization agent (ethylene glycol) in a polyesterification reaction that is promoted by mild heating [141,142]. As this step involves temperature, a volume reduction also occurs, and a more viscous (polymeric) solution is obtained at the end of this process. One example that has been widely employed in laboratories and industry is the Pechini methodology, in which a rigid polyester matrix, which contains single or multi-cations that are uniformly distributed, is formed [141]. 

In essence, all sol-gel or polymeric networks are broken during thermal treatment. The cations are able to react and form metal oxide nanomaterials, either in powder form, core-shell structures, or thin-film [143]. The PP route has advantages, such as its practicality, adaptability, stoichiometric control, and facility for obtaining polymeric solutions or gels of different viscosities [138,144,145]. However, despite this great versatility in preparing nanomaterials in various physical forms and chemical compositions, this flexibility can also compromise or benefit optoelectronic applications, as will be discussed in this section.

To design photoelectrodes for PEC devices, as the epitaxial growth of thin films onto substrates, a systematic control over the polymeric precursor solution, its deposition conditions, and the thermal treatment parameters are crucial to performance. One of the invariable points observed in different films prepared from the PP method was morphology, consisting of elongated grains with different diameters and lengths [76,146,147,148,149,150]. For hematite films, XRD analysis has shown that the PP approach led to the formation of the hematite phase with preferential orientation in the [110] axis vertical plane that possesses a strong anisotropy in electronic conductivity, which is similar to nanorod or nanowire morphology. As stated previously, this preferential orientation should facilitate the electron collection for an improved PEC response. 

**Figure 5:**
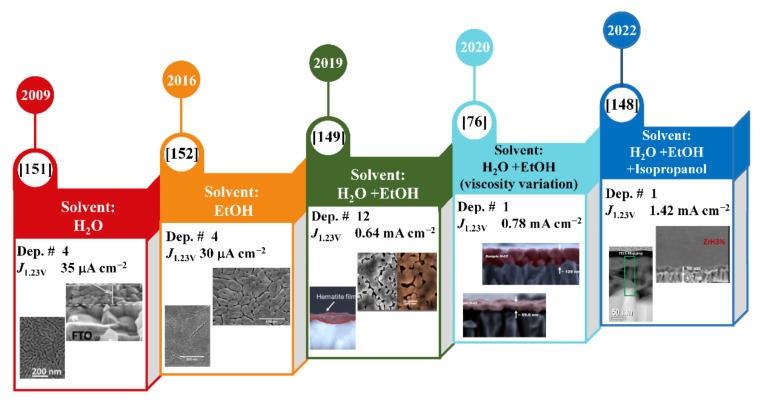
Timeline of significant breakthroughs in the synthesis of spin-coated hematite photoanodes by the polymeric precursor method from 2009 to 2022. The deposition number is represented by *Dep #*, and the J_1.23V_ represents the photocurrent density obtained at 1.23 V vs. reversible hydrogen electrode (RHE). Reprinted from Reference [151], Copyright 2009; Reference [152], Copyright 2016; Reference [149], Copyright 2019; Reference [76], Copyright 2020; Reference [148], Copyright 2022, with permission from Elsevier.

Nevertheless, the photoelectrochemical activity is derived from multiple inherent aspects of the material, among which optical absorption needs to be addressed. Hematite films require over 400 nm thickness for effective photon absorption (~95% of 550 nm) of the sunlight intensity [38]. A single deposition of the polymeric solution might result in a film thickness of ~40–100 nm, depending on the viscosity and thermal treatment employed. This means that at least four depositions must take place to obtain the optimized films. The initial works on hematite films from PP were based on such grounds, including the group’s results, shown in Figure 5. As expected, our studies showed that the photocurrent improved with the film thickness that resulted from multiple depositions and/or by incorporating modifiers, such as Si^4+^, Zn^2+^, or Sn^4+^. Still, the maximum values were only 35 μA cm^−2^, which was 0.3% of the theoretically predicted efficiency [150,151,152]. Some explanations for the low response were discussed, particularly the photogenerated charge recombination processes that were driven by the modifier segregation at the grain boundaries, which might act as recombination sites, and at the hematite–electrolyte interface, creating surface states. Furthermore, the poor hematite adherence onto the FTO was due to stress release in the thermal treatment that reduced the electron injection to the back-contact.

The thermal treatment is indispensable for the films prepared from the PP method to obtain the oxide phase and to activate the material for further application. When a polymeric solution is deposited onto the irregular substrate surface, the flexible polymer creates a “soft carpet” and takes its surface shape. During the thermal treatment, the elimination of organic compounds and the material crystallization induce a rearrangement in the conformal feature, causing a decrease in the adhesion due to the creation of empty regions (non-contact) and significant lattice strains at the hematite–FTO interface [148,153]. Thus, mismatches between the substrate and the thin film are common and not exclusive to hematite, creating a “dead layer” at the back-contact interface [148,154]. The solvent exchange and viscosity adjustment of the PP solution were hypothesized to mitigate this issue from a different mechanism of conversion and rearrangement of the polymeric layer to oxide during thermal treatment, as proven in Muche et al. [149]. Reducing the water content and including ethanol in the PP solution has allowed multiple depositions of hematite thin films with benchmark performance (from μA cm^−2^ to mA cm^−2^). The greater adhesion of hematite on the FTO improved the electron injection to the back contact. However, the successive deposition increases the number of grain–grain interfaces that would block the electron pathway by increasing the charge recombination in bulk. So, hematite performance could be improved even further if these issues are overcome.

A later approach of varying the viscosity of the precursor solution with ethanol demonstrated that similar and higher photocurrent density could be obtained for both pristine and Sn-modified hematite monolayers, with grains oriented in the [110] plane [76]. Interestingly, although this single deposition simplifies the fabrication process and reduces the number of grain interfaces found in the previous study, the thickness remained at 130 nm, which is still far from the optimal value for hematite films. Moreover, the hematite adhesion to the substrate diminishes as a side effect. The know-how of hematite synthesis from the PP method and the impacts of those abovementioned modifications, encouraged the development of another approach that involved the incorporation of a different modifier (Zr^4+^) and the solvent exchange for a mixture of alcohols [148]. The zirconium content in the polymeric precursor solution slightly modified the hematite grain morphology and resulted in more particles connected to the FTO, to the detriment of the empty regions obtained previously. As a result, the photocurrent response reached values that were 40 times higher than those in our first report (Figure 5). 

Like hydrothermal synthesis, hematite films prepared by the PP method have shown photocurrent values that are lower than the theoretical ones. Furthermore, some drawbacks to overcome are the solid–solid FTO/hematite interface, the low absorption coefficient, and hematite’s intrinsic defects. For this reason, in-situ doping and segregation strategies have been utilized to increase the PEC performance in both methodologies, using element modifiers, such as Sn, Zn, Si, Ge, and Ti [73,76,148,155]. It is worth mentioning that, compared to columnar nanostructured films, the surface area of PP-derived films is smaller; therefore, a small content of modifiers would probably be required to reach the best performance. Moreover, the strategies that lead to element segregation have shown more promising results, associated with a lower energy barrier between hematite grains, which enhances the electronic transport of bare hematite [156]. However, when the modifier segregates at the hematite surface exposed to the electrolyte, surface states are created, demanding more overpotential to drive the water oxidation reaction. This behavior was observed when Sn^4+^ and Zr^4+^ were employed as modifiers of the PP solution [76,148]. More importantly, PEC measurements in the presence of hole scavengers have shown that the intrinsic defects or the creation of new ones significantly affect the electrodes. The addition of cocatalysts or passivating agents is often needed to optimize the photoresponse. 

**Figure 6:**
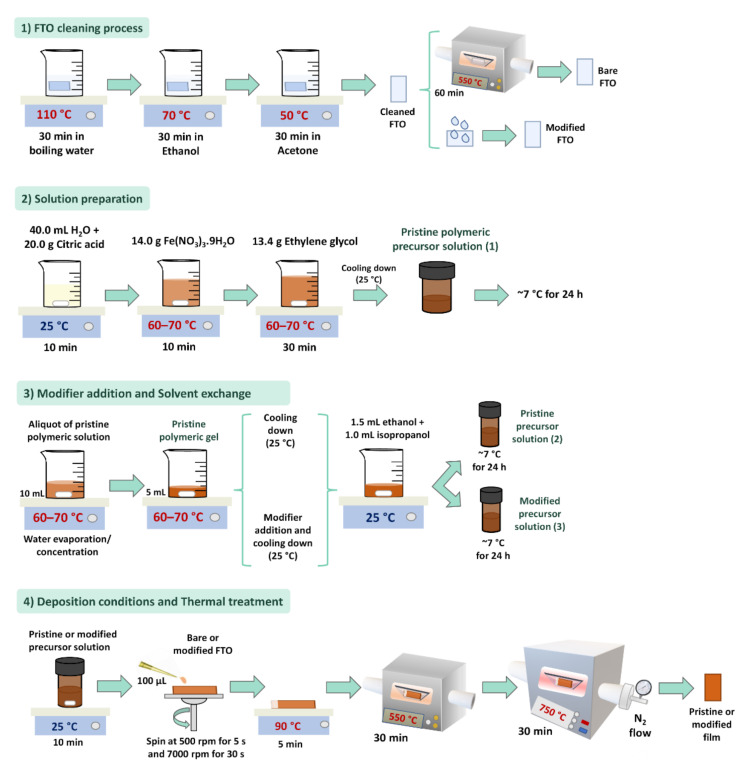
Main steps of the thin film preparation from the polymeric precursor method, optimized to receive different modifiers, minimizing the electron loss at the interfaces. Fluorine-doped tin oxide (FTO) glass substrate corresponds to the commercial substrate; modified FTO represents any chemical surface modification, such as an underlayer coating prior to photocatalyst deposition; solution preparation shows the common hematite synthesis as an example of nanostructured oxide that can be obtained from polymeric precursor route; and modifier addition denotes any element different from those present in the solution preparation that is employed for some optoelectronic improvement.

The porous nature of the films, beneficial for catalysis, can also bring some drawbacks that are associated with shunting recombination in the area where the substrate is in contact with the electrolyte. To avoid this problem, the addition of an isolating underlayer of a few nanometers—such as Ga_2_O_3_ [157], TiO_2_ [158], SnO_2_ [159], or even the deposition of a top polymeric layer [61]—has been successfully explored. Our group’s latest work incorporated the underlayer approach to the improved PP methodology [160]. Besides the latest advances in PP methodology and its potential application for an industrial-scale implementation, its optimization still represents a challenge related to interface engineering from nanoscale (grain–grain interface and FTO–grain interface) to microscale (semiconductor–electrolyte interface). The development of a simple method that accomplishes all requirements and improves the overall efficiency by reducing the deficiencies in each interface and by minimizing the photogenerated losses is still the holy grail in the design of efficient PEC devices. To start walking in this direction, we explored different strategies that led to the best results, bringing them together in a synthesis protocol to obtain nanostructured oxides that were adaptable to receive modifications that acted in synergy toward performance improvement, as illustrated in Figure 6. 

The first and most essential step involves the FTO cleaning process (1), which removes any surface impurities. The as-cleaned substrate is directly used in some syntheses, such as hydrothermal. However, the compact film formed by the PP synthesis is more susceptible to poor adherence on the inhomogeneous FTO surface. Better surface uniformity was obtained when the substrate was subjected to thermal treatment, improving the interaction of the polymeric solution further deposited. Therefore, a 1 h thermal treatment at 550 °C is adopted for the cleaned substrates (bare FTO), except when it is desirable to cover it with an underlayer (modified FTO), because the underlayer covering from simple methodologies usually involves a thermal treatment. A typical PP solution preparation (2) begins with the dissolution of citric acid in the solvent, which is optimized as water for the hematite synthesis. Then, a metal precursor is added in the 1:3 ratio of citric acid. After complete homogenization, the system is heated at 60–70 °C, and ethylene glycol is added to initiate the polymerization. A pristine polymeric stock solution is obtained after 30 min and can be directly used to prepare hematite films, or a concentration step can be performed. By reducing 50% of the initial volume, a pristine polymeric gel is obtained, in which modifier incorporation can be achieved with a rapid homogenization. The pristine or modified polymeric gel is allowed to cool down to 25 °C, at which point a viscosity adjustment is made by adding ethanol and isopropanol (1.5:1). The resulting pristine or modified precursor solutions need to be stored (in a refrigerator, 7 °C) for at least 24 h before use to obtain reproducible films. The spin-coating deposition (3) from 100 µL of the precursor solution and rotation conditions of 500 rpm for 5 s and 7000 rpm for 30 s ensure uniform coverage for solutions with different viscosities. A mild drying in a hot plate initiates the solvent evaporation and film adhesion to the substrate, which is completed in the following air thermal treatment, at 550 °C; this eliminates the polymeric chain and promotes the crystallization of the nanostructured oxide. The hematite film is then treated in a nitrogen atmosphere at 750 °C to activate it for PEC applications. 

The above-mentioned protocol exemplified for hematite film preparation can be extended to various materials and applications. Even so, as the focus of this perspective is on nanomaterials in alternative energy production, it was necessary to consider the suitable film adhesion and thermal treatment to activate it for PEC. In this sense, we hope that this methodology can serve as a basis for other researchers to prepare thicker materials, maintaining the good optoelectronic properties achieved so far. 

## 4. Outlook

The development of different approaches for hematite photoanodes has led to significant advances in the design of functional materials for PEC devices. This perspective has highlighted the progress that has been achieved in preparing hematite photoanodes by hydrothermal and sol-gel synthesis, which has impacted their PEC performance. Diverse strategies have been devoted to understanding the charge-carrier mechanism of the hematite photoanode, which is directly influenced by the preparation technique. 

Hydrothermal synthesis shows excellent potential in preparing functional materials, and hematite is not an exception. Creating successful design methodologies must consider the solid–liquid interfacial tension, the surface free energy, the ionic strength, the point of zero charge (PZC), and the thermodynamic equilibrium. By employing hydrothermal synthesis, it is possible to control morphology and particle sizes, as well as the growth orientation and the crystal structure, which directly influences the PEC performance. Experimental results have shown that the morphology depends on the hydrothermal temperature, the ionic strength controller and the concentration of the dopant. Among all the possible resultant morphologies, 1D nanorod morphology has shown remarkable results, which reinforce its ability to overcome hematite electronic drawbacks. The nanorod length and diameter can be tuned by modifying the synthesis time. However, the PEC results of these arrays of hematite photoanodes have demonstrated that other strategies must be employed to improve these performances, such as element doping and segregation, thermal treatments, or cocatalyst addition. Creating new pathways to control these modifications in a scalable and straightforward methodology might be the main challenge in considering hydrothermal synthesis for industrial applications. 

The systematic control over the stoichiometry in the polymeric precursor approach enables the synthesis of modified hematite heterostructures with excellent reproducibility. Although this approach is widely applied for coating manufacturing, which implies a simple technology transfer for PEC applications, the PEC performance of hematite photoanodes is relatively low due to the high number of interfaces that provoke electron losses. Although different reports have shown the same grain morphology that can vary in size according to the employed solvent or by adding an element, our group has demonstrated that a particular strategy of adding an element can also modify the grain shape and improve the charge collection. As the adhesion to the substrate directly affects the PEC response, this point represents one of the biggest issues to address going forward. Our latest efforts to successfully mitigate those problems aligned with identifying the atomic position of the elemental addition and its role in the overall hematite performance is the way towards achieving the predicted benchmark efficiency.

Indeed, the high surface area, film thickness, and nanostructures provided by hydrothermal synthesis are some critical advantages for designing highly efficient photoanodes, which is reflected in the current photocurrent benchmark. Although the benchmark for the hematite photoanodes synthesized by the PP route is inferior to those obtained by the hydrothermal route, the recent strategies summarized in this perspective have opened the possibility of increasing their PEC response. In both cases, we believe that a fine-tuned control of the thermal treatment and a deeper understanding of its impact on the defect chemistry and substrate integrity can help us to go further. A broader scope of the surface states is essential to creating effective strategies for designing photoanode electrodes. In this sense, using in-situ and in-operando techniques could be the key. In the race to develop functional photoelectrodes for low-cost commercial PEC devices, hydrothermal and polymeric precursor routes are both the leading fabrication methods that scientific and industrial communities must pursue over the coming years.

## Data Availability

Not applicable.
